# A95 ASSESSMENT OF BIRTH COHORT SCREENING OF CHRONIC HEPATITIS C IN COLORECTAL CANCER SCREENING PATIENTS IN BRITISH COLUMBIA

**DOI:** 10.1093/jcag/gwac036.095

**Published:** 2023-03-07

**Authors:** R Kaviani, F Y Chou, C B He, V Marquez

**Affiliations:** 1 Internal Medicine; 2 Faculty of Medicine; 3 Gastroenterology, University of British Columbia, Vancouver, Canada

## Abstract

**Background:**

Birth cohort screening of chronic hepatitis C (CHC) is recommended in British Columbia since 2018 for baby boomers born between 1945 to 1964, with an estimated provincial prevalence of 2.31%. Though there remained a gap in care following anti-hepatitis C positivity, resulting in reflexive ribonucleic acid (RNA) testing provincially. Dual screening of CHC in patients referred to colorectal (CRC) screening programs can provide an opportunity to link patients with healthcare professionals to ensure appropriate follow-up.

**Purpose:**

We aimed to assess the uptake of CHC screening amongst CRC screening patients after the release of British Columbia’s birth cohort guidelines, both pre and post-COVID-19 pandemic.

**Method:**

A retrospective review of patients referred to a CRC screening program in Vancouver from October 1st to December 31st, 2019, and December 1st – 31st, 2021, was performed. Collected data included demographics, liver disease history, and co-infection rates with hepatitis B (HBV) or human immunodeficiency virus (HIV). Dates of first-time hepatitis C antibody, RNA and viral load testing were gathered. Descriptive statistics were used to identify the proportion of screening and prevalence of CHC.

**Result(s):**

A total of 553 patients were referred for colonoscopy to the CRC screening program, of whom 458 (82.8%) patients were born between 1945 to 1964, and 273 (n=49%) were female. Among the 250 (45.2%) patients screened for CHC, 4 (0.72%) had positive anti-hepatitis C, all of whom were baby boomers. In 2019, 44% (n=183) of patients were screened for CHC; 78.7% (n=144) were screened before colonoscopy referral. In 2020, 48.6% (n=67) of patients were screened for CHC; 100% of cases were screened before colonoscopy referral.

**Conclusion(s):**

Birth cohort screening of CHC is underutilized in British Columbia. Dual screening of CHC at the time of referral to CRC screening provides a practical approach to linking patients to healthcare.

**Please acknowledge all funding agencies by checking the applicable boxes below:**

None

**Disclosure of Interest:**

None Declared

